# Loneliness, Depression, and Anxiety Experienced by the Israeli Population During the First COVID-19 Lockdown: A Cross-sectional Survey

**DOI:** 10.5041/RMMJ.10449

**Published:** 2021-10-25

**Authors:** Jason Brafman, Robert Lubin, Revital Naor-Ziv, Sarah Rosenberg, Tzvi Dwolatzky

**Affiliations:** 1 The Ruth & Bruce Rappaport Faculty of Medicine, Technion–Israel Institute of Technology, Haifa, Israel; 2 Department of Criminology, Bar-Ilan University, Ramat Gan, Israel; 3 Keep Olim, Society for the Advancement of Immigrants to Israel, Jerusalem, Israel; 4 Geriatric Unit, Rambam Health Care Campus, Haifa, Israel

## INTRODUCTION

The presence of loneliness, depression, and anxiety is known to be associated with increased overall morbidity.^1^,^2^ These conditions may occur as individual syndromes or in combination. There is a clear need to recognize these conditions when they occur and to provide patients with appropriate care and support. From a public health perspective, it is important to evaluate both the prevalence and the epidemiological risk factors associated with these syndromes. For example, approximately 50% of mental health disorders have been shown to begin during the mid-teenage years.^3^ As such, mental health screening should specifically target the younger population since, while these disorders frequently manifest early on in adolescence, intervention and treatment are not usually initiated until years later.^4^ At times of stress and crisis, mental health disorders in younger individuals are often more pronounced. This may reflect an exacerbation of pre-existing depression or anxiety, may manifest the transformation of a pre-clinical mental disorder into a symptomatic one, or may represent a risk factor for the development of a mental health condition *de novo*.

The coronavirus 2019 (COVID-19) pandemic is certainly the greatest challenge facing countries, populations, economies, and health care services in the last 20 years. The stresses resulting from this pandemic place an additional burden on the mental health of people of all ages.^4^–^6^ Traditionally, government health funding has not emphasized planning for the mental health consequences of such major public health challenges.^7^ Generally, mental health resources are scarce, with minimal emphasis on mental health screening, and services for populations who are at higher risk for mental health morbidity are inadequate. Many studies have already noted the important impact that COVID-19 has had on mental health.^8^–^13^

This study sought to evaluate the differences in the prevalence of self-reported symptoms of depression, anxiety, and loneliness between younger and older generations at the time of the COVID-19 pandemic. Since the younger generation is generally less accustomed to facing and dealing with adversity and illness, we hypothesized that adolescents and younger adults would have a higher prevalence of depression, anxiety, and loneliness as compared to the older generations. It must be emphasized that this survey was conducted during the first COVID-19 lockdown that occurred in Israel from mid-March 2020 to early-May 2020. This was a time when businesses were closed, individuals and families were isolated at home with very limited social contact, and feelings of fear and panic were fueled by the electronic media.

## METHODS

Our study included participants of the age groups representing Generation Z (16–23 years), Millennials (24–39 years), Generation X (40–55 years), Baby Boomers/Maturists (>56 years). The survey was distributed to students and faculty of both the Ruth & Bruce Rappaport Faculty of Medicine at the Technion–Israel Institute of Technology and of Bar-Ilan University, and was posted on social media sites such as Facebook and WhatsApp. The survey was available in Hebrew, English, and Spanish. A notification was sent to those who expressed an interest in completing the survey confirming that their responses would be confidential, and an “opt-out” option was available for those who did not want their responses used for research. The study was approved by the Ethics Review Board of Bar-Ilan University (Ref. 21 May 2020), and the researchers had access only to anonymized data.

The Four-item Patient Health Questionnaire (PHQ-4), a brief screening tool for depression and anxiety,^14^ was made available to respondents who had the option of completing it in Hebrew, English, or Spanish. The four items are four questions regarding symptoms of anxiety or depression or both. The PHQ-4 was chosen for screening for depression and/or anxiety based on its attributes of high sensitivity and specificity as well as brevity.^14^ The sum of the four items of the PHQ-4 yields a composite score ranging from 0 to a maximum of 12. For the purpose of our study a score of 2 or less on the PHQ-4 was considered to be negative, while a score of 3 or more was defined as a positive screen for depression/anxiety. Participants were also asked to complete other self-reporting measurements and to provide demographical information as well as answer a screening question about feelings of loneliness. The responses on screening for loneliness were “never,” “sometimes,” “more than half the time,” or “almost every day.” The wording was slightly changed from the classic PHQ-4 for a more appropriate grammatical fit to a context that applied only during the COVID-19 lockdown in Israel from March to May 2020 as well as for cognitive ergonomics. An identical translation was done previously by Lowe et al.^15^ and Zhao et al.^16^ Also, the original language of the survey was Spanish; as such, our word choice fit better with a direct translation from the Spanish, maintaining consistency between languages. The Hebrew translation was likewise grammatically and syntactically consistent. A response of “more than half the time” or “almost every day” was defined as a positive screen for loneliness, while a response of “never” or “sometimes” was defined as negative.

As mentioned, individuals constituting Generation Z are at increased risk for mental health issues, and as such may be less resilient in coping with the stresses of challenging global events such as the current pandemic. Based on this premise, we designated a cutoff of those born in 1997 and later in order to distinguish those constituting Generation Z from older participants.

## RESULTS

**Figure 1:**
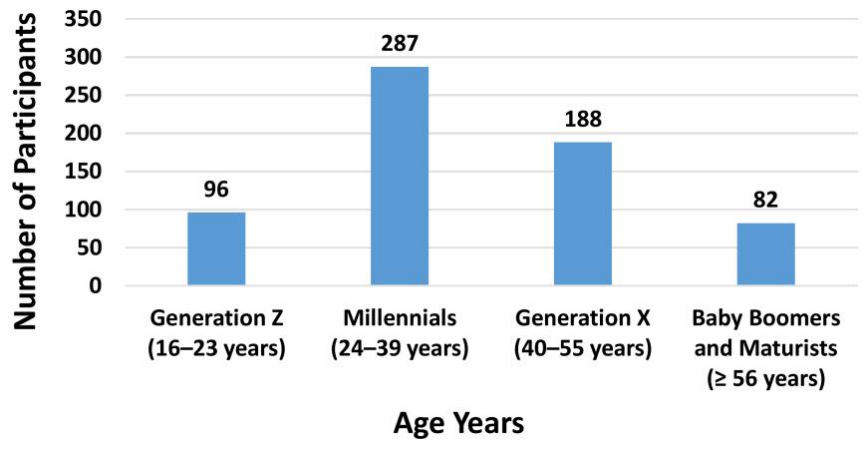
Age Distribution of Respondents (*n*=653).

A total of 665 individuals responded to the survey, of which 653 included their age in their response and were included in the data analysis (range, 16–85 years; mean=38). The age distribution of the study group is presented in Figure 1. Of the 653 responses included in the analysis, 406 responded in Hebrew, 195 in English, and 52 in Spanish.

Relevant elements of the survey for analysis were: age, and if the respondent had experienced “loneliness,” “little interest or pleasure in doing things,” “weariness, depression, or hopelessness,” “nervousness or tension,” and “inability to stop or control worrying thoughts.” The last four aforementioned elements constitute the PHQ-4. The four possible answers for the PHQ-4 and loneliness were “never,” “sometimes,” “more than half the time,” or “almost every day,” scored as 0, 1, 2, or 3, respectively. Results are presented in Table 1. The cutoff for depression, anxiety, and loneliness was “more than half the time” or worse. We found that self-reported loneliness was highest among Generation Z. Interestingly, self-reported depression and anxiety was almost the same for Generation Z and Mil-lennials (77% and 78%, respectively), though it was also reported by Generation X and Baby Boomers.

**Table 1:** Responses to the Survey with Regard to Loneliness and the PHQ-4 According to Age Group.

Survey	Generation Z (16–23 years) (*n*=96)	Millennials (24–39 years) (*n*=287)	Generation X (40–55 years) (*n*=188)	Baby Boomers and Maturists (≥56 years) (*n*=82)
Self-reported Loneliness				
Never, *n* (%)	33 (34.3)	127 (44.2)	118 (62.8)	50 (61.0)
Sometimes, *n* (%)	31 (32.3)	87 (30.3)	42 (22.3)	27 (32.9)
More than half the time, *n* (%)	13 (13.5)	35 (12.2)	18 (9.6)	2 (2.4)
Almost every day, *n* (%)	19 (19.8)	38 (13.2)	10 (5.3)	3 (3.7)

Self-reported PHQ-4				
Negative (score 0–2), *n* (%)	22 (23.0)	63 (22.0)	58 (30.9)	27 (32.9)
Positive (score ≥3), *n* (%)	74 (77.0)	224 (78.0)	130 (69.1)	55 (67.1)

PHQ-4, 4-Item Patient Health Questionnaire.

Statistical analysis using the one-sided ANOVA test revealed statistical significance for loneliness (*F* ratio value: 9.42961, *P* value: <0.00001), but none for the PHQ-4 depression and anxiety data (*F* ratio value: 2.43138, *P* value: 0.064084). Post hoc Tukey HSD testing also yielded significant differences for loneliness as shown in Table 2.

**Table 2:** Post Hoc Tukey HSD for Loneliness Results.*

Pairwise Comparisons	*Q* Value	*P* Value
Generation Z : Millennials	2.22	0.40
Generation Z : Generation X	5.19	0.001
Generation Z : Baby Boomers/Maturists	7.66	<0.001
Millennials : Generation X	2.97	0.15
Millennials : Baby Boomers/Maturists	5.44	<0.001
Generation X : Baby Boomers/Maturists	2.47	0.30

* After one-sided ANOVA showed statistical significance in loneliness.

## DISCUSSION

In this self-reported survey, we found that younger respondents experienced a greater incidence of feelings of loneliness at the time of the first lockdown in Israel during the COVID-19 pandemic as compared to older respondents. While there was a trend for experiencing a greater feeling of depression and/or anxiety in these younger respondents, this difference was not significant.

Our age cutoff levels were chosen based on the definitions of generational cohorts, namely Generation Z, Millennials, Generation X, Baby Boomers, and Maturists. Our premise was that younger individuals were fitter, healthier, and more adaptive to change and would thus be more able to adjust to the stress of a pandemic/lockdown. Although we do recognize the possibility of unrecognized mental health issues in adolescents and young adults as mentioned earlier, the age group constituting Generation Z and Millennials generally is healthier and less likely to experience major physical illness or disability as compared to the older population. As such, we may expect these younger generations to be more resilient at the time of social stress and upheaval. Our finding of a greater feeling of loneliness in the younger generational cohorts is thus interesting. While we have not identified similar published studies looking at the generational effects of loneliness and mental health at the time of the COVID-19 pandemic, our results conform with recent reports in the media.^17^,^18^ This finding raises important issues that should be addressed, such as disrupted rhythms of life and the social effects of lockdown.

There are several limitations to this study. The survey was distributed via universities, making individuals without a connection to a university less likely to have received an invitation to participate in the survey. In addition, although we received a good number of responses to the survey, a larger sample size that is more representative of the Israeli population would clearly improve the generalizability of our findings. The survey was available in English, Hebrew, and Spanish, and we did not compare responses based on language. Clearly, those of different backgrounds, particularly immigrants compared to native Israelis, may be more prone to psychological distress and loneliness as they try to adapt to a new environment at the time of a pandemic. While the survey was conducted specifically in the narrow time frame of the first COVID-19 lockdown in Israel, conducting the survey over a longer time period may have provided a greater insight into the subsequent development of loneliness, depression, or anxiety. Our finding that self-reported loneliness was highest in Generation Z should be regarded with caution since the number of respondents in this group was relatively small compared to the other groups. Also, the feeling of loneliness in Generation Z may be related to the fact that they are generally less accustomed to facing and dealing with adversity and other factors, and thus the association with the COVID-19 lockdown may not be causative.

Our findings are interesting and raise important issues. Adolescents and young adults are more prone to loneliness, and the association of loneliness and mental health symptomatology, especially anxiety and depression, with the ongoing COVID-19 pandemic should be further investigated. In fact, the symptoms of psychological stress were high in all age groups. These symptoms should be recognized and treated on a personal level, and appropriate public health measures should be implemented. With the continuing pandemic worldwide, the long-term effects of limitations such as social isolation should be carefully assessed, mapping of loneliness and psychological stress should include all age groups, and health care organizations and policy makers should initiate widespread policies of support and stress management.

## Abbreviations

COVID-19: coronavirus 2019
PHQ-4: Four-item Patient Health Questionnaire.
